# Atypical brain structural connectivity and social cognition in childhood maltreatment and peer victimisation

**DOI:** 10.1186/s12888-024-05759-3

**Published:** 2024-04-16

**Authors:** Lena Lim, Lia Talozzi, Henrietta Howells

**Affiliations:** 1 Singapore Institute for Clinical Sciences (SICS), Agency for Science, Technology and Research (A*STAR), Singapore, Singapore; 2 Department of Child & Adolescent Psychiatry, Institute of Psychiatry, Psychology & Neuroscience (IoPPN), King’s College London, London, UK; 3 Neurology and Neurological Sciences, Stanford University, California, USA; 4 Laboratory of Motor Control, Department of Medical Biotechnology and Translational Medicine, University of Milan and Humanitas Research Hospital, Milan, Italy

**Keywords:** Early-life stress, Childhood trauma, Peer bullying, DTI, Theory of mind

## Abstract

**Background:**

Childhood maltreatment (CM) is associated with neurobiological aberrations and atypical social cognition. Few studies have examined the neural effects of another common early-life interpersonal stressor, namely peer victimisation (PV). This study examines the associations between tract aberrations and childhood interpersonal stress from caregivers (CM) and peers (PV), and explores how the observed tract alterations are in turn related to affective theory of mind (ToM).

**Methods:**

Data from 107 age-and gender-matched youths (34 CM [age = 19.9 ± 1.68; 36%male], 35 PV [age = 19.9 ± 1.65; 43%male], 38 comparison subjects [age = 20.0 ± 1.66; 42%male] were analysed using tractography and whole-brain tract-based spatial statistics (TBSS).

**Results:**

At the whole-brain level using TBSS, the CM group had higher fractional anisotropy (FA) than the PV and comparison groups in a cluster of predominantly limbic and corpus callosal pathways. Segmented tractography indicated the CM group had higher FA in right uncinate fasciculus compared to both groups. They also had smaller right anterior thalamic radiation (ATR) tract volume than the comparison group and higher left ATR FA than the PV group, with these metrics associated with higher emotional abuse and enhanced affective ToM within the CM group, respectively. The PV group had lower inferior fronto-occipital fasciculus FA than the other two groups, which was related to lower affective ToM within the PV group.

**Conclusion:**

Findings suggest that exposure to early-life stress from caregivers and peers are differentially associated with alterations of neural pathways connecting the frontal, temporal and occipital cortices involved in cognitive and affective control, with possible links to their atypical social cognition.

**Supplementary Information:**

The online version contains supplementary material available at 10.1186/s12888-024-05759-3.

## Background

Childhood maltreatment (CM) is common worldwide with paediatric prevalence of 13–36% [1]. It is significantly associated with first onsets of various psychiatric disorders including anxiety, depression and PTSD [2]. It has been further proposed that the psychopathological outcomes associated with CM may be mediated by the disruption of neural underpinnings [3].

Studies have reported that individuals exposed to CM exhibited impaired attention, emotion and reward processing [4–7], along with atypical error processing [8, 9] and social cognitive functioning [10]. Theory of Mind (ToM) is a key component of social cognition essential for human interactions and survival. Mental state decoding, or affective ToM, which requires various social-perceptual skills to identify emotional expressions in others [11], may be particularly affected in individuals exposed to CM. Maltreated youths growing up in abusive settings may be more sensitive and proficient at decoding emotional states of caregivers’ in order to deal with potential imminent threats [12]. Notably, some earlier findings have been confounded by psychiatric comorbidities [13–15]. For instance, CM was associated with higher mental-state identification accuracy when controlling for dissociation [13], while lower accuracy was associated with higher dissociation and depressive symptoms [15]. A recent review and meta-analysis on the association between CM and social cognition, including ToM in particular, shows mixed results in individuals with affective disorders [16]. This underlines the importance of further examining ToM in maltreated individuals specifically in the absence of any psychopathology.

CM is associated with grey matter volume (GMV) aberrations in several relatively late-developing brain regions, particularly the orbitofrontal cortex (OFC) [17, 18] and temporal lobes [19, 20], as well as the visual cortex [20, 21]. Meta-analytical studies have further reported that CM is associated with GMV reductions in prefrontal cortex (PFC)-limbic [22] and OFC-limbic-temporal and inferior frontal regions that mediate top-down affect and cognitive control, respectively [23]. In comparison, fewer studies have examined white matter (WM) aberrations in CM. Brain regions do not function independently; rather, they communicate through a complex system of short-and long-range WM tracts [24] that regulate the speed and timing of activation across neural networks, which are essential for optimal performance of higher-order social-cognitive tasks that rely on integrated information processing [25]. Furthermore, stress can affect WM tract development, as corticosteroids can suppress the final mitosis of glial cells necessary for myelination [26]. Moreover, WM has a protracted postnatal developmental timeline with different trajectories; specifically, corticolimbic tracts and their connections undergo protracted maturation into the third decade of life [27]. Hence, these corticolimbic tracts may be particularly vulnerable to the neurotoxic impact of early-life trauma, especially during certain sensitive periods.

Fractional anisotropy (FA) is a DTI-derived metric that may reflect aspects of membrane integrity and myelin thickness, where atypical levels in either direction can signal dysfunction depending on the brain region [28]. Studies have found that CM is associated with aberrations in several large WM tracts, particularly the inferior fronto-occipital fasciculus (IFOF) [29–33], inferior longitudinal fasciculus (ILF) [31, 32], uncinate fasciculus (UF) [29, 34, 35], anterior thalamic radiation (ATR) [32, 36], corpus callosum [30, 32, 36] and superior longitudinal fasciculus (SLF) [30, 34]; thereby suggesting that CM is associated with widespread WM microstructural aberrations predominantly evident in the neural pathways linking fronto-limbic and occipital visual cortices that are presumably involved in conveying and processing the (aversive) experience.

Emerging research underscores the importance of parent/caregiver and peer relationships on brain development of young people. Besides caregivers, peers are also crucial for a child’s socio-emotional development as they learn and develop critical social skills though their interactions with peers outside the familial settings. However, peers can also be a significant source of interpersonal stress during childhood. Peer victimisation (PV) is characterised by repetitive aggressive behaviour such as overt confrontation, ostracism, relational and reputational aggression engaged by an individual or group with the intention to cause harm [37]. It is a serious global issue with paediatric prevalence of 20–30% [38]. Several large-scale studies also reported that PV has deleterious developmental and mental health consequences including poor school performance and development of psychopathology [39], and suggest the possibility of an underlying neurobiological substrate for PV [40].

The field has made much progress in documenting the neurobiological correlates of CM, but research investigating neural alterations in PV has been relatively limited and the few DTI studies on PV reported mixed findings. For instance, PV is associated with decreased FA in the right posterior corona radiata in young adults [41], but with increased FA in the right medial lemniscus and left posterior corona radiata in depressed adolescents [42], and with increased FA in the corpus callosum, bilateral corona radiata and right sagittal stratum in children [43]. However, another study found no significant association in bullied adolescents at risk of psychosis [44]. Also, it remains unclear if the structural differences observed were specifically associated with PV, in the absence of psychiatric comorbidities.

Furthermore, CM and PV may have unique and/or additive effects on the development of maladaptive cognitive structures and psychological maladjustment. For instance, a retrospective study of young adults found that parental emotional abuse (controlling for peer verbal victimisation) predicted dysfunctional attitudes but not cognitive style; while peer verbal victimisation (controlling for parental emotional abuse) predicted cognitive style but not dysfunctional attitudes [45]. A longitudinal prospective study of community youths reported that harsh parenting and PV, taken together or separately, predicted changes in youths’ negative and positive self-cognitions and depressive symptoms, and harsh parenting exhibited incremental importance over-and-above PV on youths’ self-cognitions [46]. Hence, given that CM and PV may have differential effects on mental health outcomes [45, 46], and maltreated children are at increased risk of subsequent victimisation by peers possibly via altered neurocognitive functioning [47], it is imperative that studies examine PV in the absence of prior exposure to CM.

Tractography facilitates the reconstruction of 3D trajectories of specific WM tracts and probes their microstructure, which allows a more detailed analysis of specific subpopulations of fibres and indirect volumetric indices [48]. As the standard “tract-averaged” approach may obscure variation in DTI metrices within tracts [49], we also conducted an exploratory along-tract analysis of FA to provide more fine-grained analysis. Tract-based spatial statistics (TBSS) permits a whole-brain analysis of WM in a voxel-wise manner, which allows the identification of WM differences in specific regions beyond a priori defined tracts [50]. Hence, we used these complementary methods to examine atypical WM tracts in CM and PV.

The aim of the present study was to examine the common and specific associations between WM tract aberrations and childhood interpersonal stress from caregivers (CM) and peers (PV) by conducting tract-specific and whole-brain analyses in non-clinical/community youths free from psychopathology, medications and drug abuse. To examine the specificity of the association with the nature of early-life interpersonal stress, we controlled for the timing and duration of exposure to aversive caregiving and peer bullying as well as the number of recent stressors experienced. We excluded childhood sexual abuse so that the CM is compatible with the PV group as bullying from peers is usually non-sexual and also because it has different effects on brain structure [51] and different behavioural and psychiatric consequences [52] relative to other abuse and neglect experiences. Scholars have argued that childhood sexual abuse is associated with experiences unique to sexual victimisation; for example, traumatic sexualisation, betrayal and stigmatisation may affect victims of sexual abuse more profoundly and/or differently than victims of other abuse experiences [53, 54].

Given that CM is associated with GMV deficits in OFC-limbic-temporal and occipital visual regions along with aberrations in the WM tracts connecting these regions, we hypothesised that the limbic tracts (UF, ATR, ILF and IFOF) implicated in socio-affective functioning may be particularly affected in individuals exposed to early-life stress from caregivers or peers. The PV group may exhibit tract aberrations to a lesser extent than the CM group since peer-related stress were perceived to be less stressful than parent-related stress in youths [55]. We also investigated alterations in regions beyond our priori-defined tracts with a whole-brain TBSS analysis.

## Methods

### Participants

Potential participants were recruited from the community via advertisement in social clubs/organisations and on social media. Exclusion criteria were childhood sexual abuse, current and/or past psychiatric diagnoses, drug abuse, psychotropic medications, bullying perpetration, neurological abnormalities, brain injuries and learning disabilities. First, we conducted a thorough pre-screening interview to assess the exclusion and inclusion criteria, where potential participants were first explicitly asked if they had any of the exclusion characteristics listed, and were excluded if they met any of the exclusion criteria. Hence, those who perpetrated bullying (regardless of whether they were victimized or not) were excluded at this stage. Next, severity of the early-life stressful experiences were assessed using the Childhood Trauma Questionnaire (CTQ) [56], Revised-Peer Experiences Questionnaire (RPEQ) [57] and European Cyberbullying Intervention Project Questionnaire (ECIPQ) [58]. Information on the age onset and duration of the early-life stressful experiences were collected with the two questions: “How old were you when you first experienced the harsh treatment from the caregiver(s) or peer(s)?” and “For how long did you experience the harsh treatment from the caregiver(s) or peer(s)”. Inclusion criteria for the CM group were non-sexual maltreatment from caregivers scoring above the cut-off for moderate severity on at least one of the CTQ subscales, but did not experience bullying from peers (scoring “Never”/“Once or twice” on the RPEQ and ECIPQ). Inclusion criteria for the PV group were frequently bullied by peers (scoring at least “a few times”/“once a month” on the RPEQ and ECIPQ), but without a history of maltreatment from caregivers (scoring below the cut-offs for none/low severity on all the CTQ subscales). The comparison group did not experience maltreatment from caregivers and bullying from peers (scoring below the respective cut-offs for the RPEQ/ECIPQ and CTQ). Interested volunteers that were deemed suitable were next invited to participate in the study, while those found unsuitable were notified and their information was deleted immediately at this stage. A total of 108 youths (35 CM, 35 PV and 38 comparison) (age range:17–21 years) participated.

All participants and their guardians provided written informed consent and were reimbursed $80 for their time. All procedures involving human subjects were approved by the Nanyang Technological University’s Institutional Review Board and all MRI scans were reviewed by a neuro-radiologist.

### Procedure

The study consisted of a face-to-face interview and an MRI session that took place either on the same day or on a different day within a 1-week period. During the interview session, all participants completed the following: DSM-5 Level-1 Cross-Cutting Symptom Measure and the Kiddie Schedule for Affective Disorders and Schizophrenia Present and Lifetime Version (KSADS-PL) interviews for psychopathology, Strengths and Difficulties Questionnaires (SDQ) [59], Beck’s Depression Inventory (BDI) [60], Beck’s Anxiety Inventory (BAI) [61] and the Negative and Positive Affect Scale (NAPAS) [62]. The Childhood Experience of Care and Abuse (CECA) interview [63] was used to corroborate the CTQ and provide additional information on the age onset and duration of the maltreatment experiences. IQ was assessed using the Wechsler Abbreviated Scale of Intelligence (WASI) [64]. Socioeconomic status (SES) was measured with six items (on parental educational level, housing size and type) from the Family Affluence Scale [65]. Recent stressful life events (RSLE) was assessed using common stressors adapted from the Life Event Questionnaire for Adolescents [66], where participants rated the 12-month incidence and distress level of each stressor. A total RSLE score was calculated by summing the number of items that were rated as quite or very stressful. In the present study, the internal consistency of the questionnaires ranged from 0.88 to 0.93. Lastly, Reading the Mind in the Eyes” Test (RMET) [67], a widely used computer-based behavioural task, was used to evaluate affective ToM (SI).

### MRI data acquisition

A standardised MRI protocol was acquired using a 3 T Siemens MAGNETOM Prisma scanner for all participants, including a structural T1-weighted image (MPRAGE, 1x1x1mm^3^) and diffusion-weighted images (b-value = 2000s/mm^2^, 64 diffusion-weighted directions). Diffusion data were pre-processed using automatic pipelines adopting FSL (https://fsl.fmrib.ox.ac.uk/fsl/fslwiki) and MRtrix3 (https://www.mrtrix.org) functions (SI).

### Tractography

A fully automatised pipeline was developed for the virtual dissection of the following limbic tracts: UF, ATR, ILF and IFOF. ROIs were defined in the MNI-152 space adapting FSL-AutoPtx tract ROIs (https://fsl.fmrib.ox.ac.uk/fsl/fslwiki/AutoPtx). A two-ROI seed-target approach was used for all tracts, and exclusions regions were defined to avoid false connection reconstructions from nearby WM tracts. The UF was dissected, including streamlines running between the anterior temporal pole and the fronto-orbital cortex; the ATR between the thalamus and dorsolateral PFC; the ILF between the anterior temporal lobe and the parieto-occipital cortex; and the IFOF between the occipital cortex and the frontal lobe (Fig. S2).

Tractography ROIs were defined in the MNI-152 space. A two-step ROI transformation was adopted. First, a non-linear transformation was computed to register the MNI-152 T1 space to the subject’s T1-weighted image (FSL-fnirt function). Second, the subject’s T1-weighted image was registered to his/her diffusion-weighted MRI (FSL-epi_reg function). The obtained transformation fields were applied to transform the tractography ROIs first onto the subject native T1 and subsequently onto the diffusion-weighted space where the tractography reconstructions were performed. The diffusion-weighted signal was modelled using constrained spherical deconvolution, and probabilistic tractography was performed (tckgen ifod2-Mrtrix3). A 10% threshold to the maximum of connectivity was applied for all tracts to filter false-positive connections due to the probabilistic tractography algorithm. This tractography protocol, which has been previously compared to other tractography methods for the arcuate fasciculus [68] and tested in the presence of brain tumours [69–71], is used for other WM tracts in the current study.

Group variability maps were generated and displayed as 90% of shared variability across subjects to show the reliability of the obtained tractography results. That is, when the trajectory of spurious fibres significantly diverged from the 90% of shared variability, the fibre was flagged as a false positive artefact and removed for the participant. The visual inspection of the tracts and manual cleaning was performed by L.T. with FSLeyes in editing mode. Notably, only a few spurious fibres were corrected. Such a robust WM delineation was due to the stringent threshold used on tractography results and tuned for each tract on the estimated maximum connectivity.

The along-tract method used parameterises the tract volume surface considering its 3-dimensional mesh as a connectivity matrix [68]. Subsequently, the Laplacian operator allowed the along-tract segment division using its first eigenvalue evaluated on the connectivity matrix. The along-tract division was performed in the MNI space within the WM core using an anterior-posterior tract segmentation order. To overcome individual subject variability and artefactual connections when WM fibres are branching towards cortical terminations, the along tract analyses were restricted to the tract WM core, i.e., the compact WM bundle before the fanning toward cortical projections [68, 72]. In the MNI space, coordinate limits were imposed for the voxel location in the MNI-152 space, y-coordinate increasing posteriorly-anteriorly and the x-coordinate from right to left. Specifically, for the ILF, y_mi*n* = 35 and y_max = 66; for the IFOF, y_min = 27 and y_max = 78; for the UF arching, only a y_max = 80 was needed; and for the ATR, y_min = 57 and y_max = 78, and x_min = 30 and x_max = 75. Subsequently, the tract WM cores were registered back to the native DTI space, where DTI measure statistics were evaluated to avoid signal smoothing due to the intensity interpolation of registration methods. The number of segments for which each tract was divided was based on an average calculation of tract volume across participants. The number of along-tract segments corresponded to the average tract volume in the MNI space divided by 3cm^3^ (Fig. S3).

Group differences in whole-tract volume and DTI metrics were performed using ANOVA with Statistical Package for the Social Sciences (SPSS) version 26 (SPSS Inc., Chicago, IL, USA). Our primary diffusivity analyses focused on the FA values as majority of previous studies reported FA measures significantly differing in early-life stress [41, 42]. We hypothesised that changes in early life may be captured more directly in FA contrast; nevertheless, we also included information on mean diffusivity (MD), radial diffusivity (RD) and axial diffusivity (AD) (Table S1).

### TBSS

Voxelwise statistical analysis of the FA data was carried out using TBSS [50], part of FSL [73]. First, FA images were created by fitting a tensor model to the raw diffusion data using FDT, and then brain-extracted using BET [74]. All participants’ FA data were then aligned into a common space using the nonlinear registration tool FNIRT [75, 76], which uses a b-spline representation of the registration warp field [77]. Next, the mean FA image was created and thinned to create a mean FA skeleton which represents the centres of all tracts common to the group. Each participant’s aligned FA data was then projected onto this skeleton and the resulting data fed into voxelwise cross-subject statistics. 5000 permutations of a GLM were applied. The statistical threshold was set at *p* < 0.05, fully corrected for multiple comparisons using threshold-free cluster enhancement (TFCE).

### Statistical analysis

Statistical analyses were carried out using SPSS26. Demographic and clinical data were analysed with ANOVA and post-hoc *t*-tests adjusted for multiple comparisons, while χ2 and Fishers-exact tests were used for categorical demographic variables. RSLE, age onset and duration of early-life stress exposure were included as covariates in all analyses to avoid confounding the effect of early-life stress with recent stressors and the impact of timing and duration of early-life stress exposure, so that the CM and PV groups differ only on the nature (caregivers vs. peers) of their early-life stress. As the ROIs examined were defined a priori and identified independently based on earlier studies, no adjustment for multiple comparisons was made. Finally, Pearson correlations were used to explore associations between brain metrices and RMET performance (response accuracy), early-stress (CTQ, RPEQ/ECIPQ) and psychological (BDI, BAI, SDQ) measures within each group. Given the exploratory nature of the correlational and along-tract tractography analyses, results were not adjusted for multiple comparisons.

## Results

### Sample characteristics

All participants reported no current and/or past psychiatric disorders, which was further validated with the DSM-5 Level-1 Cross-Cutting Symptom Measure and the KSADS-PL interviews. They also reported no head trauma injuries or loss of consciousness. One participant from the CM group had to be excluded due to MRI motion artefacts. Hence, the final sample consisted of 107 participants (34 CM, 35 PV and 38 comparison).

There were no group differences in age, gender, IQ, ethnicity and SES (Table 1). As anticipated, the CM and PV groups scored significantly higher than the comparison group on: BDI, BAI, NAPAS negative affect, RSLE and SDQ emotional and total difficulties (*p* < 0.01), but lower than the comparison group on NAPAS positive affect (*p* < 0.001). However, their depression and anxiety scores were still within normative range below the cut-offs for moderate severity on the BDI and BAI, respectively. The CM and PV groups did not differ from each other except on SDQ peer problems where the PV group scored the highest. The CM group had significantly lower age of onset and longer duration of early-stress exposure than the PV group (*p* < 0.001) (Table 1).

**Table 1:** Demographic characteristics of 34 youths exposed to childhood maltreatment, 35 youths exposed to peer victimisation and 38 comparison participants

Characteristic	Childhood Maltreatment group (*n* = 34)		Peer Victimisation group (*n* = 35)		Comparison group (*n* = 38)		Analysis^d,e^		Group Comparisons^a^
	Mean	SD	Mean	SD	Mean	SD	*F*(2,104)	*p*	
**Age (years)**^**b**^	19.9	1.68	19.9	1.65	20.0	1.66	0.07	ns	**–**
**IQ**	103.9	10.1	103.1	8.26	102.5	7.24	0.27	ns	**–**
**Socioeconomic status (SES)**^**c**^	15.4	3.90	17.1	3.67	16.3	3.31	1.29	ns	–
**Recent stressful life events scale** **(RSLE)**	1.21	1.04	1.40	1.33	0.37	0.71	10.0	< 0.001	PV, CM > C
**Beck’s Depression Inventory (BDI)**	8.76	6.97	10.1	8.76	3.16	3.69	11.0	< 0.001	PV, CM > C
**Beck’s Anxiety Inventory (BAI)**	7.74	8.41	9.0	9.96	2.47	3.47	7.45	0.001	PV, CM > C
**Negative and Positive Affect Scale (NAPAS):**									
*Negative affect*	11.9	3.93	12.3	5.72	8.42	2.86	8.91	< 0.001	PV, CM > C
*Positive affect*	17.6	4.75	17.9	4.46	23.1	3.13	20.9	< 0.001	PV, CM > C
**Strengths and Difficulties Questionnaire (SDQ):**									
*Emotional problems*	3.91	2.04	4.14	2.55	2.34	1.67	7.99	0.001	PV, CM > C
*Conduct problems*	1.79	1.55	1.89	1.45	1.18	1.06	2.89	(0.06)	–
*Hyperactivity*	4.06	2.47	3.60	2.44	2.76	2.21	2.78	(0.07)	–
*Peer problems*	2.38	1.63	3.11	1.73	1.58	1.39	8.58	< 0.001	PV > CM > C
*Prosocial*	7.41	2.11	7.71	1.93	8.53	1.47	3.57	0.03	CM, PV < C
*Total difficulties score*	12.2	5.39	12.7	5.95	7.87	4.40	9.40	< 0.001	PV, CM > C
**Childhood Trauma Questionnaire (CTQ):**									**CTQ Severity Classification**
*Physical abuse*	14.3	3.94	6.80	1.49	5.21	0.53	144.3	< 0.001	CM > PV, C
*Emotional abuse*	17.7	3.40	8.34	2.26	5.82	1.09	237.1	< 0.001	CM > PV, C
*Physical neglect*	10.1	2.58	6.54	2.09	5.68	1.12	47.4	< 0.001	CM > PV, C
*Emotional neglect*	17.4	3.30	9.86	3.40	6.87	2.28	115.8	< 0.001	CM > PV, C
**Revised Peer Experience Questionnaire (RPEQ):**									
*Relational victimisation*	1.33	1.45	8.66	2.67	0.53	0.83	218.8	< 0.001	PV > CM, C
*Overt victimisation*	1.00	1.48	10.5	4.75	0.05	0.23	146.4	< 0.001	PV > CM, C
*Reputational victimisation*	1.03	1.31	9.49	2.29	0.16	0.50	398.8	< 0.001	PV > CM, C
**European Cyberbullying Intervention Project Questionnaire** **(ECIPQ)**	1.06	1.54	10.4	7.06	0.71	1.06	60.2	< 0.001	PV > CM, C
							***F*(1,67)**	***p***	
**Age at onset of CM or PV (years)**	6.71	2.64	10.2	2.39	–	–	33.9	< 0.001	CM < PV
**Duration of CM or PV (years)**	8.79	3.79	4.21	1.82	–	–	41.3	< 0.001	CM > PV
	**N**	**%**	**N**	**%**	**N**	**%**	**χ**^**2**^	***p***	**Group Comparisons**
**Gender (Males)**	12	36	15	43	16	42	0.80	ns	–
**Ethnicity**^**f**^**:**							6.81	ns	–
*Chinese*	30	88	27	77	36	94			
*Malay*	3	9	3	9	1	3			
*Indian*	1	3	5	14	1	3			

^a^*CM* childhood maltreatment group, *PV* peer victimisation group, *C* comparison group

^b^The age range was 17–21 years

^c^The SES total score ranges from 6 to 26, with higher values indicating higher status

^d^Tests adjusted for multiple comparisons

^e^The values in parentheses are marginally statistically significant

^f^The Fisher’s Exact Test was used

### Affective ToM

For RMET performance, the CM group had higher response accuracy in the emotional-state condition than the PV group (*F*(1,64) = 5.37, *p* = 0.024) only. There were no significant group differences in the age/gender condition and in task reaction time in both conditions (Table 2).

**Table 2:** Group performance on the Reading the Mind in the Eyes Task (RMET)

*RMET*Measure	Childhood Maltreatment group (*N* = 34)		Peer Victimisation group (*N* = 35)		Comparison group (*N* = 38)		Group Comparisons^a,b^								
							*CM* vs *C*			*PV* vs *C*			*CM* vs *PV*		
	Mean	SD	Mean	SD	Mean	SD	*F*(1,67)	*p*		*F*(1,68)	*p*		*F*(1,64)	*p*	
**Emotional-state Condition**															
% correct	74.2	8.11	70.9	8.62	69.5	8.50	1.33	ns	–	1.09	ns	–	5.37	0.024	CM > PV
Mean RT^c^ (ms)	3723	490	3723	694	3829	506	0.10	ns	–	1.30	ns	–	0.008	ns	–
**Age/Gender Condition**															
% correct	80.5	8.12	82.6	7.24	85.9	7.19	2.82	ns	–	1.17	ns	–	0.008	ns	–
Mean RT (ms)	2789	526	2762	482	2801	484	0.001	ns	–	0.63	ns	–	0.03	ns	–

^a^*CM* childhood maltreatment group, *PV* peer victimisation group, *C* comparison group

^b^Group differences in RMET measures were conducted with number of recent stressful life events, age onset and duration of early-life stress exposure as covariates

^c^*RT* reaction time

### Tractography

**Fig. 1:**
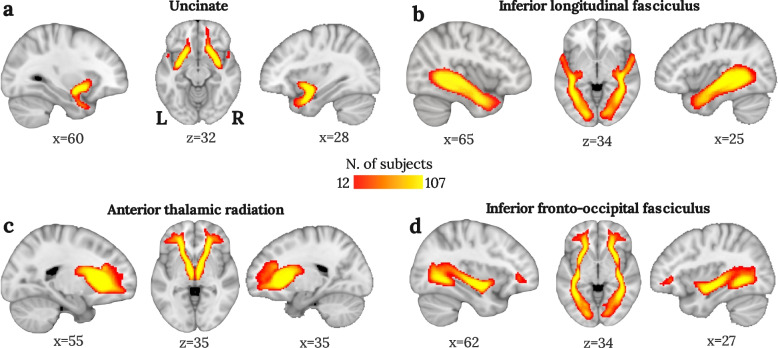
Tractographic group variability maps onto the MNI-152 space of the Uncinate Fasciculus (UF), Anterior Thalamic Radiation (ATR), Inferior Longitudinal Fasciculus (ILF) and Inferior Fronto-Occipital fasciculus (IFOF) tracts*.* Group variability maps are shown according to the (**a**) uncinate fasciculus, (**b**) inferior longitudinal fasciculus, (**c**) anterior thalamic radiation, and (**d**) inferior fronto-occipital fasciculus. Intensity scales range from 10% of the group overlap (*N* = 12 subjects, red-coloured) to 100% (*N* = 107 subjects, yellow-coloured). The reported coordinates are according to the voxel localization in the MNI-152 space (2 mm resolution)

The tractography pipelines allowed tract dissection for all participants. The resulted group variability maps are reported in Fig. 1.

The CM group had significantly smaller right ATR tract volume than the comparison group (Cohen’s d = 0.20, *p* = 0.021) and smaller right UF tract volume than the PV group (Cohen’s d = 0.91, *p* = 0.021), who did not differ from the comparison group. Smaller ATR tract volume was associated with higher CTQ emotional abuse (r = − 0.42, *p* = 0.014, 95% CI [− 0.66, − 0.09]) and CTQ total (r = − 0.35, *p* = 0.046, 95% CI [− 0.61, − 0.01]) scores within the CM group as well as with higher BAI (r = − 0.46, *p* = 0.004, 95% CI [− 0.68, − 0.17]) within the comparison group (Fig. S4).

**Fig. 2:**
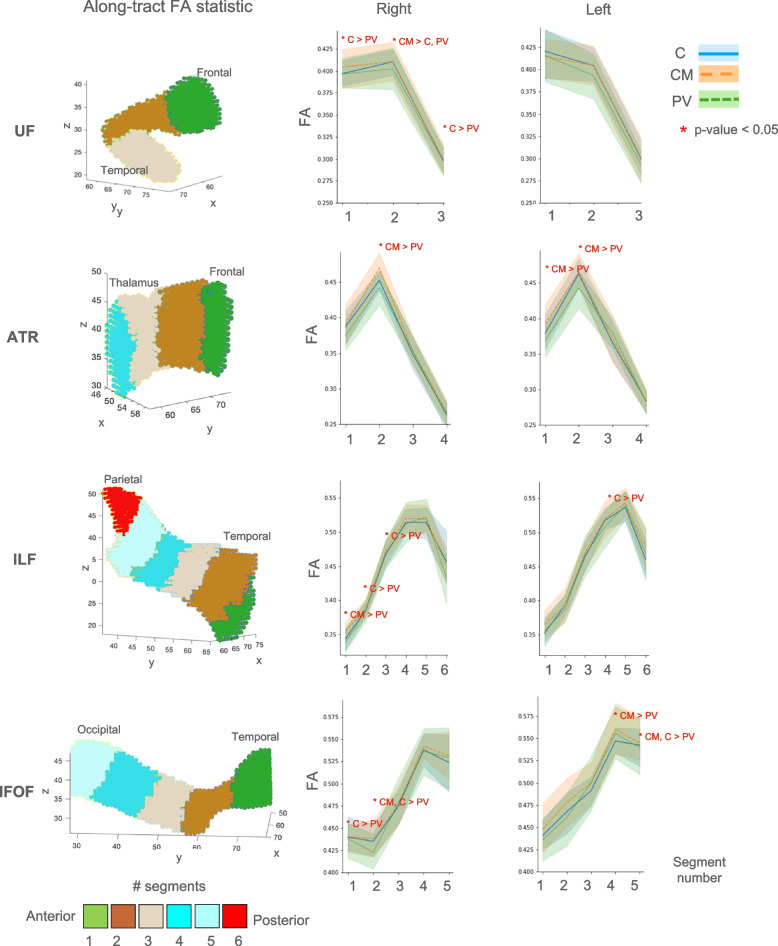
Along-tract FA profiles and plot statistics of the Uncinate Fasciculus (UF), Anterior Thalamic Radiation (ATR), Inferior Longitudinal Fasciculus (ILF) and Inferior Fronto-Occipital fasciculus (IFOF) tracts. For each tract, a 3D scatter plot shows the voxel localisation coloured according to the respective along-tract segment number. The scatter plot coordinates (x, y, and z) are reported according to voxel localisation in the MNI-152 space (2 mm resolution). Along-tract FA profiles are shown for the right and left tract results. On the abscissa axis the number of segments and on the ordinate axis the median FA within each segment for the comparison (C), childhood maltreatment (CM) and pear victimisation (PV) groups. Shaded areas correspond to the group interquartile distribution (25th–95th percentile). Red asterisks indicate group differences with *p*-value < 0.05

At the microstructural level, the CM group had significantly higher FA in right UF (Cohen’s d = 0.21, *p* = 0.019) than the comparison group as well as higher FA in right UF (Cohen’s d = 0.21, *p* = 0.035), left IFOF (Cohen’s d = 0.46, *p* = 0.011) and marginally in left ATR (Cohen’s d = 0.53, *p* = 0.061) than the PV group, where higher FA in left ATR was associated with higher accuracy in the RMET emotional-state condition (r = 0.39, *p* = 0.024, 95% CI [0.06, 0.64]) within the CM group, while lower FA in left IFOF (r = 0.40, *p* = 0.016, 95% CI [0.08, 0.65]) and right UF (r = 0.39, *p* = 0.021, 95% CI [0.07, 0.64]) were associated with lower RMET emotional-state accuracy within the PV group (Fig. S5). The PV group had lower FA in right UF (Cohen’s d = 0.05, *p* = 0.008) and bilateral IFOF (Left: Cohen’s d = 0.16, *p* = 0.011; Right: Cohen’s d = 0.05, *p* = 0.011) and higher FA in bilateral ILF (Left: Cohen’s d = 0.01, *p* = 0.045; Right: Cohen’s d = 0.12, *p* = 0.025) than the comparison group, thereby suggesting that the increased right UF FA could be maltreatment-related while the decreased left IFOF FA could be bully-related (Table 3, Fig. 2). Furthermore, lower left IFOF FA was associated with greater SDQ emotional problems (r = − 0.35, *p* = 0.030, 95% CI [− 0.61, − 0.04]) within the comparison group (Fig. S6).

**Table 3:** Group differences in tract volume and fractional anisotropy of the Uncinate Fasciculus (UF), Anterior Thalamic Radiation (ATR) Inferior Longitudinal Fasciculus (ILF) and Inferior Fronto-Occipital Fasciculus (IFOF) tracts

Tract Measurement	Childhood Maltreatment group (*n* = 34)		Peer Victimisation group (*n* = 35)		Comparison group (*n* = 38)		Group Comparisons^a,b,c^								
							*CM* vs *C*			*PV* vs *C*			*CM* vs *PV*		
	Mean	SD	Mean	SD	Mean	SD	*F*(1,67)	*p*		*F*(1,68)	*p*		*F*(1,64)	*p*	
**Right UF**															
Tract Volume (mm^3^)	3423	463	3937	651	3702	645	0.09	ns	**–**	0.001	ns	**–**	5.64	0.021	CM < PV
Fractional Anisotropy:															
*Tract-averaged*	0.372	0.021	0.367	0.026	0.368	0.017	5.81	0.019	CM > C	7.48	0.008	PV < C	4.66	0.035	CM > PV
*Segment 1*	0.399	0.036	0.399	0.036	0.395	0.026	2.20	ns	**–**	4.82	0.032	PV > C	0.38	ns	**–**
*Segment 2*	0.415	0.032	0.403	0.036	0.407	0.026	5.10	0.027	CM > C	2.94	(0.09)	(PV < C)	5.31	0.024	CM > PV
*Segment 3*	0.301	0.030	0.299	0.026	0.300	0.020	0.085	ns	**–**	6.10	0.016	PV < C	0.74	ns	**–**
**Left UF**															
Tract Volume (mm^3^)	2991	482	3256	555	3115	548	0.45	ns	**–**	0.18	ns	**–**	0.63	ns	**–**
Fractional Anisotropy:															
*Tract-averaged*	0.379	0.028	0.375	0.032	0.380	0.023	0.48	ns	**–**	2.24	ns	**–**	0.99	ns	**–**
*Segment 1*	0.413	0.046	0.413	0.045	0.420	0.034	0.45	ns	**–**	0.23	ns	**–**	0.56	ns	**–**
*Segment 2*	0.406	0.032	0.396	0.038	0.402	0.028	2.24	ns	**–**	1.23	ns	**–**	2.75	ns	**–**
*Segment 3*	0.297	0.036	0.292	0.030	0.298	0.025	1.93	ns	**–**	0.42	ns	**–**	0.003	ns	**–**
**Right ATR**															
Tract Volume (mm^3^)	5154	1096	5390	1048	5353	918	5.56	0.021	CM < C	0.018	ns	**–**	0.006	ns	**–**
Fractional Anisotropy:															
*Tract-averaged*	0.369	0.022	0.357	0.020	0.362	0.018	0.78	ns	**–**	1.71	ns	**–**	1.60	ns	**–**
*Segment 1*	0.395	0.043	0.376	0.036	0.387	0.034	0.62	ns	**–**	0.24	ns	**–**	1.73	ns	**–**
*Segment 2*	0.471	0.034	0.445	0.036	0.454	0.026	0.19	ns	**–**	0.66	ns	**–**	5.09	0.027	CM > PV
*Segment 3*	0.356	0.038	0.353	0.027	0.349	0.031	2.91	(0.09)	(CM > C)	0.07	ns	**–**	0.006	ns	**–**
*Segment 4*	0.267	0.021	0.260	0.021	0.264	0.022	3.24	(0.07)	(CM > C)	0.003	ns	**–**	0.31	ns	**–**
**Left ATR**															
Tract Volume (mm^3^)	5113	898	5348	752	5200	857	3.11	ns	**–**	0.26	ns	**–**	0.28	ns	**–**
Fractional Anisotropy:															
*Tract-averaged*	0.377	0.022	0.365	0.023	0.369	0.020	0.60	ns	**–**	1.01	ns	**–**	3.65	(0.06)	(CM > PV)
*Segment 1*	0.388	0.046	0.370	0.031	0.381	0.042	0.95	ns	**–**	1.40	ns	**–**	5.71	0.020	CM > PV
*Segment 2*	0.466	0.044	0.446	0.044	0.463	0.030	0.02	ns	**–**	1.57	ns	**–**	4.01	0.050	CM > PV
*Segment 3*	0.372	0.041	0.373	0.040	0.365	0.041	2.90	(0.09)	(CM > C)	0.07	ns	**–**	0.03	ns	**–**
*Segment 4*	0.285	0.026	0.282	0.020	0.283	0.027	0.10	ns	**–**	0.30	ns	**–**	0.04	ns	**–**
Tract Volume (mm^3^)	6508	1629	6943	1568	7630	1859	0.12	ns		2.11	ns	**–**	0.002	ns	**–**
Fractional Anisotropy:															
*Tract-averaged*	0.456	0.020	0.453	0.028	0.450	0.021	1.17	ns	**–**	5.28	0.025	PV > C	0.73	ns	**–**
*Segment 1*	0.355	0.029	0.337	0.025	0.343	0.028	0.02	ns	**–**	1.98	ns	–	5.07	0.028	CM > PV
*Segment 2*	0.390	0.024	0.382	0.033	0.387	0.022	0.68	ns	**–**	15.9	0.001	PV < C	2.06	ns	**–**
*Segment 3*	0.471	0.022	0.469	0.033	0.470	0.025	0.32	ns	**–**	9.09	0.004	PV < C	0.48	ns	**–**
*Segment 4*	0.519	0.029	0.512	0.033	0.514	0.027	0.61	ns	**–**	1.29	ns	**–**	1.43	ns	**–**
*Segment 5*	0.519	0.030	0.521	0.035	0.511	0.035	0.001	ns	**–**	1.04	ns	**–**	0.02	ns	**–**
*Segment 6*	0.458	0.054	0.433	0.054	0.453	0.059	0.004	ns	**–**	1.29	ns	**–**	2.10	ns	**–**
Tract Volume (mm^3^)	6435	1881	6666	1600	6883	1530	0.16	ns		1.07	ns	**–**	0.004	ns	**–**
Fractional Anisotropy:															
*Tract-averaged*	0.468	0.024	0.461	0.029	0.461	0.020	0.19	ns	**–**	4.17	0.045	PV > C	1.32	ns	**–**
*Segment 1*	0.364	0.032	0.354	0.026	0.353	0.029	0.02	ns	**–**	0.10	ns	**–**	0.58	ns	**–**
*Segment 2*	0.392	0.031	0.385	0.032	0.393	0.024	0.07	ns	**–**	2.35	ns	**–**	1.12	ns	**–**
*Segment 3*	0.475	0.040	0.465	0.037	0.465	0.028	0.83	ns	**–**	1.02	ns	**–**	0.75	ns	**–**
*Segment 4*	0.522	0.040	0.508	0.039	0.515	0.025	0.06	ns	**–**	10.2	0.002	PV < C	3.06	(0.08)	(CM > PV)
*Segment 5*	0.538	0.034	0.540	0.034	0.535	0.031	0.001	ns	**–**	1.03	ns	**–**	0.72	ns	**–**
*Segment 6*	0.476	0.053	0.469	0.055	0.455	0.060	0.10	ns	**–**	0.003	ns	**–**	2.05	ns	**–**
Tract Volume (mm^3^)	7016	845	6869	980	7023	1236	2.72	ns		0.001	ns	**–**	0.03	ns	**–**
Fractional Anisotropy:															
*Tract-averaged*	0.490	0.181	0.484	0.027	0.485	0.019	0.23	ns	**–**	6.87	0.011	PV < C	1.37	ns	**–**
*Segment 1*	0.443	0.026	0.438	0.032	0.441	0.026	1.75	ns	**–**	6.67	0.012	PV < C	1.15	ns	**–**
*Segment 2*	0.441	0.033	0.427	0.035	0.439	0.029	1.60	ns	**–**	4.34	0.041	PV < C	5.27	0.025	CM > PV
*Segment 3*	0.475	0.030	0.477	0.032	0.475	0.028	1.78	ns	**–**	3.04	(0.08)	(PV > C)	0.13	ns	**–**
*Segment 4*	0.543	0.026	0.535	0.035	0.533	0.033	0.008	ns	**–**	3.54	(0.06)	(PV > C)	0.76	ns	**–**
*Segment 5*	0.532	0.040	0.529	0.048	0.521	0.044	1.88	ns	**–**	0.22	ns	**–**	0.09	ns	**–**
Tract Volume (mm^3^)	6954	1340	6836	1324	6741	1137	1.18	ns		0.07	ns	**–**	0.36	ns	**–**
Fractional Anisotropy:															
*Tract-averaged*	0.510	0.025	0.497	0.031	0.501	0.016	1.83	ns	**–**	6.92	0.011	PV < C	6.88	0.011	CM > PV
*Segment 1*	0.447	0.042	0.433	0.037	0.443	0.028	0.99	ns	**–**	1.90	ns	**–**	3.11	(0.08)	(CM > PV)
*Segment 2*	0.478	0.040	0.463	0.040	0.470	0.036	2.78	ns	**–**	0.87	ns	**–**	1.84	ns	**–**
*Segment 3*	0.500	0.035	0.500	0.038	0.494	0.029	1.23	ns	**–**	2.53	ns	**–**	0.26	ns	**–**
*Segment 4*	0.561	0.033	0.554	0.043	0.549	0.027	0.03	ns	**–**	2.36	ns	**–**	4.96	0.029	CM > PV
*Segment 5*	0.543	0.045	0.540	0.048	0.541	0.035	0.20	ns	**–**	5.67	0.020	PV < C	4.49	0.038	CM > PV

^a^*CM* childhood maltreatment group, *PV* peer victimisation group, *C* = comparison group

^b^The values in parentheses are marginally statistically significant

^c^Group differences in tract volume and fractional anisotropy were conducted with number of recent stressful events, age onset and duration of early-life stress exposure as covariates

Along-tract analysis of FA values further revealed specific maltreatment-related increased FA in the middle insular cortex portion of right UF (segment 2) as well as bully-related reduced FA in the posterior part of left IFOF (segment 5) and anterior part of right IFOF (segment 2), where lower FA of right anterior IFOF was related to greater SDQ peer problems (r = − 0.48, *p* = 0.003, 95% CI [− 0.70, − 0.18]) (Fig. S6) within the PV group. Group differences between CM and PV groups occurred mainly in the anterior part of bilateral ATR and right ILF (Table 3, Fig. 2).

### TBSS

**Fig. 3:**
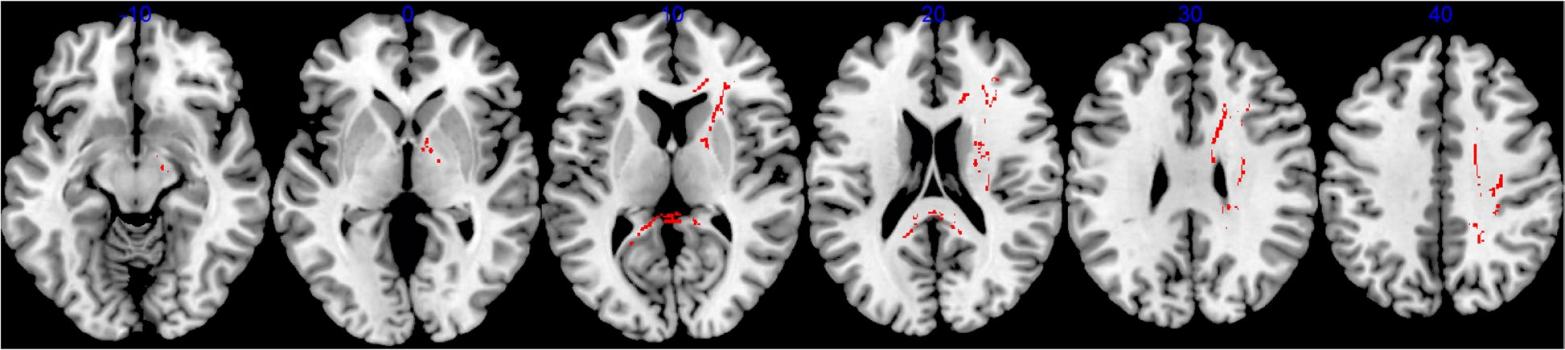
Whole-brain TBSS analysis of significantly increased FA values in the childhood maltreatment group relative to the comparison group (*p* < 0.05, TFCE-corrected)

The CM group had significantly higher FA than the comparison group in a cluster comprising the corpus callosum (splenium, body and genu), bilateral cingulum bundle and corticospinal tracts along with the right ATR, IFOF, ILF, UF, SLF and anterior corona radiata (Table 4, Fig. 3). In addition, we tested if spread WM alterations were present in response to the early-stress events despite a tract-specific distribution. Thus, mean FA values were next extracted for planned post-hoc analysis with the PV group, controlling for RSLE, age onset and duration of early-life stress exposure. The CM group also had significantly higher FA than the PV group (*F*(1,64) = 8.40, *p* = 0.005) who did not differ from the comparison group, thereby suggesting that the higher FA observed may be specific to the maltreatment experience.

**Table 4:** Cluster of increased white matter fractional anisotropy in the childhood maltreatment group compared with the comparison group (*p* < 0.05, TFCE-corrected^a^)

	MNI-152 Coordinates^b^ (mm)	Cluster size	*p*^*c,d*^
Forceps major and minor/ Bilateral Cingulum bundle/ Corticospinal tract/ Right Anterior thalamic radiation /Anterior corona radiata/ Superior longitudinal fasciculus/ Inferior fronto-occipital fasciculus/ Inferior longitudinal fasciculus/ Uncinate fasciculus	−17, −44, 7	3766	0.028

^a^*TFCE* Threshold-Free Cluster Enhancement

^b^*MNI* Montreal Neurological Institute

^c^Threshold-free cluster enhancement-corrected *p* values

^d^Group differences were conducted with number of recent stressful events, age onset and duration of early-life stress exposure as covariates

## Discussion

Early relationships with caregivers and peers are known to affect normal brain development, yet little research has compared the negative neurobiological effects associated with being maltreated by caregivers and bullied by peers. Furthermore, given that CM and PV have additive effects on mental health outcomes [47], studies should examine the impact of exposure to peer bullying in the absence of adverse caregiving and vice versa. To our knowledge, this is the first DTI study to do so in a sizable non-clinical youth sample free from psychopathology, medications and drug abuse and controlled for the number of recent stressors as well as the timing and duration of early-life stress exposure. This is essential to elucidate the effects of early-life stress from the confounding effects associated with current stressors, timing and duration of early-life stress, psychiatric comorbidities, medications and drug use [23].

Tractography analysis showed that the CM group had significantly smaller right ATR tract volume than the comparison group, which was furthermore associated with greater maltreatment severity particularly emotional abuse in maltreated individuals. At the microstructural level, the CM group had significantly higher FA in right UF than the comparison group, and higher FA in right UF, left IFOF and ATR than the PV group, who had lower FA in right UF and bilateral IFOF than the comparison group; thereby suggesting that the increased right UF FA could be maltreatment-related while the decreased left IFOF FA could be bully-related. Furthermore, the CM group had higher affective ToM performance than the PV group only, which was associated with higher left ATR FA within the CM group, and with higher FA in left IFOF and right UF within the PV group. Along-tract analysis further revealed specific maltreatment-related increased FA in the middle insular cortex portion of right UF as well as bully-related reduced FA in the left posterior IFOF and right anterior IFOF, where lower FA in right anterior IFOF was related to greater SDQ peer problems within the PV group. Lastly, TBSS analysis at the whole-brain level showed that the CM group had significantly higher FA than the other two groups in a cluster of predominantly limbic and corpus callosal tracts.

WM maturation occurs throughout childhood and adolescence in the form of increasing FA and do not reach plateaus until adulthood, particularly in tracts connecting the frontal, temporal and limbic regions which follow the most protracted course [27]. Give that the WM microstructural changes possibly underlie development in affective processing and emotion regulation [78], and increases in FA has been associated with learning [79], the higher FA particularly of the limbic tracts observed in our maltreated youths may thus possibly reflect increased informational processing of affective stimuli and fear response.

The UF connects the OFC to limbic regions facilitating emotion processing and mood regulation [80]. We observed early stress-related alterations in UF at the macro-and microstructural levels. Earlier studies also reported increased UF FA in individuals exposed to CM [29, 34, 35] and traumatic events [81]. Furthermore, the microstructural integrity of the UF has been implicated in resilience after trauma where higher UF FA attenuated the association between early-life stress and later trait anxiety [35], and increased UF FA paralleled reduction of emotional symptoms and maladaptive responses to stress at follow-up [82, 83]. Similarly, a recent review of resilience in young people reported potential resilience effects in the corpus callosum where anatomical connectivity in this region is increased in resilient individuals [84]. Hence, our observed higher FA in the right UF and corpus callosum may potentially suggest some degrees of resilience in the maltreated youths. Additionally, our along-tract analysis revealed that the maltreatment-related increased FA in the right UF occurred specifically in the middle insular cortex portion, which corroborates earlier findings of insula aberrations in CM [6, 85, 86].

The ATR is part of the thalamo-fronto-striatal circuits implicated in the regulation of affective states [87]. Early-stress related alterations in ATR are observed at the macro-and microstructural levels, and its association with CM is further underpinned by a negative correlation between ATR tract volume and emotional abuse in the CM group. Additionally, the observed negative relationship between ATR tract volume and BAI within the comparison group suggests an increased vulnerability for anxiety disorders in individuals with reduced ATR tract volume. Our findings also resonate with previous studies showing higher ATR FA in individuals exposed to early neglect [36] and traumatic events [81]. Furthermore, the ATR is also part of the mentalising and mirror neural networks involved in ToM and empathy [88]. Hence, higher ATR FA, which corelated positively with affective ToM performance in our CM group, may possibly signal an enhanced recognition of caregivers’ emotional states to predict the occurrence of abuse. Thus, we hypothesise that while the adverse caregiving experience may conceivably contribute to enhanced mental-state decoding, it may also heighten the risk of developing anxiety later on, possibly via abuse-related macrostructural alterations of the ATR.

The ILF and IFOF are key components of the visual-limbic pathway mediating the visual processing of emotionally significant stimuli [89]. Altered micro-structure of these limbic-visual tracts is also consistent with earlier studies that reported increased IFOF FA [29, 30, 33] in maltreated individuals. Interestingly, we found that aberrations in the IFOF and ILF are more prominent in the PV group, where lower FA in left IFOF and right anterior IFOF were furthermore correlated with lower affective ToM and greater peer problems, respectively, within the PV group. The observed negative relationship between left IFOF FA and greater emotional problems in the comparison group also underscores the risk for psychopathology in individuals with reduced IFOF FA. A recent study found that children who were frequently victimised by peers had thicker left fusiform gyrus, which could be related to the development of social anxiety disorder given its role in processing threatening faces [40]. Thus, we propose that the observed reduced IFOF FA may reflect a delayed maturation of the IFOF pathways (and possibly associated brain regions such as the fusiform gyrus) linked to the bullied adolescents’ diminished ability to accurately decode the mental-state of peers and the ensuing immature socio-emotional processing capabilities may thus lead to greater peer problems and heightened psychopathology risk.

Although both the CM and PV groups had comparable depressive, anxiety and negative affect scores (which were still within normative ranges), it is intriguing to note that both early-life stress groups exhibited differential limbic tract aberrations with opposite direction of associations. In particular, there was a tendency for the CM group to have the highest FA of the limbic tracts followed by the comparison group and the PV group. The CM group also showed higher affective ToM capability than the PV group, which was furthermore associated with higher FA in the ATR and IFOF. Given that higher FA were found in children with higher intelligence [90] and fewer behavioural problems [91], we cautiously speculate that the maltreated youths may have accelerated maturation of the limbic pathways associated with their enhanced their affective ToM capability, while bullied youths may have delayed WM maturation and associated ToM underperformance relative to the maltreated youths. The highly stressful familial environment may compel a maltreated young person to be constantly hyper-perceptive of the caregiver’s emotional states to detect imminent threats for self-protection. It is easier for a bullied individual from a typical functional family to exit the unpleasant peer environment (e.g. through school transfer) than for a maltreated individual to leave the abusive home. Indeed, children who are bullied by peers but supported by parents often use the positive (parental support) to offset the adverse effects of the negative (peer bullying) [92]. Hence, there is a higher survival need for accelerated maturation of brain and associated socio-cognitive processing in maltreated youths than bullied youths.

The observed higher affective ToM performance and preserved IQ coupled with increased FA of the limbic tracts underscore that cognition of maltreated individuals is not generally impaired. Rather, their brains adapt and perform well on specific ecologically relevant tasks. The findings also support the stress acceleration hypothesis [93], which purports that CM may lead to accelerated maturation of core emotion circuits and behaviours as adaptations to threatening environments to promote survival. Indeed, a recent cohort study reported that traumatic events was associated with higher FA of the limbic tracts (ATR, UF, IFOF), accelerated brain maturation and better episodic memory [81]. Previous studies have also reported that youths with early caregiving stress showed more adultlike profiles of amygdala-PFC connectivity than controls [94, 95].

WM diffusivity measures such as FA have variations along the course of the WM fibres. Such variations can be due to the tract geometry (i.e., bending or more compact pathways) and are independent of pathological alterations. An along-tract method allows us to divide the WM tract into comparable segments, where the intrinsic diffusivity variations along the tract (Fig. 2) can be regressed, resulting in a more accurate statistic. Following this procedure, significant differences in the FA diffusivity measures were found to be segment-specific. The possible interpretations of these findings could be that FA alterations are ongoing in all the tract courses. Still, we can capture them only in a few segments where the subject’s statistics are comparable, e.g., in the compact WM segment #2 of the UF tract (Fig. 2). Alternatively, the FA alterations may manifest segment specific by reflecting more local WM alterations. Hence, the segment-specific FA alterations could be a more powerful lens for capturing ongoing microstructural changes. Notable, at a young age, WM structures are developing towards a final mature stage [26]. Thus, the WM architecture of the study participants (age range:17–21 years) is undergoing ongoing maturation. To overcome this source of variability, the groups were matched on age and statistical analyses controlled for RSLE, age onset and duration of exposure to early-life stress. Given that only cross-sectional data were considered, the results cannot be interpreted as individual maturation trajectories, but instead as a shared pattern of variability across young adults who underwent similar early-stress events. Finally, the current study also demonstrates the merits of incorporating both tractography and TBSS analyses. TBSS corroborated and extended the tractography findings by showing microstructural alterations beyond the hypothesised limbic tracts while tractography highlighted group differences at the macrostructural level and revealed microstructural FA differences along tract that would be obscured when averaged across the whole tract.

### Strengths and limitations

The study is cross-sectional and the findings are still correlational. We did not examine potential pre-existing brain differences and cannot ruled out the possibility that our bullied youths might have pre-existing neural, social cognitive and behavioural differences that render them more vulnerable to bullying. The use of retrospective self-report data may be subjected to recall biases. The sample population is older than most prior studies but nonetheless, it is crucial to examine the enduring effects of early-life stressors in young adulthood and beyond given a recent meta-analytic finding that CM is associated with late-life depression [96]. Finally, given the presence of crossing fibres, we caution against over-interpreting the conventional DTI-based metrics (FA, AD and RD values) as standalone biomarkers of WM microstructure [97]. Nevertheless, strengths of this study are that all participants were free from psychopathology, medications and drug abuse, their current stressors were assessed and controlled for, and the early adverse experiences were carefully substantiated by semi-structured interviews. Although the generalisability of the results may be restricted to the more resilient portion of non-clinical/ community youths despite been exposed to harsh treatment by caregivers or peers during childhood, the current findings underscore that individuals exposed to early-stress do show neural alterations even in the absence of current psychopathology.

### Supplementary Information

**Supplementary Material 1.**

## Data Availability

The datasets generated for this study are available on request to the corresponding author.
