# Supplementary material for: Atypical brain structural connectivity and social cognition in childhood maltreatment and peer victimisation
Source: BMC Psychiatry. 2024 Apr 16;24:287. doi: 10.1186/s12888-024-05759-3 (PMC11022413; doi:10.1186/s12888-024-05759-3)

**Supplementary Information**

**Methods**

***Study participants***

Participants were recruited from the general community in Singapore including schools, churches and various social clubs and organisations. All participants were English-speaking and in good health, defined as being without a history of chronic medical or psychiatric illness and free of prescription medications.

***MRI data acquisition***

MRI data were acquired using a 3T Siemens MAGNETOM Prisma (Siemens Healthcare GmbH, Germany) at NTU. We used a 64-channel head coil and a double spin-echo encoding sequence with the following parameters: TR=6000 ms, TE=90 ms, 74 slices, 64 diffusion weighted directions (b=2000 s/mm²), 9 non-diffusion-weighted volumes (b=0 s/mm²), voxel size: 2 mm^3^ isotropic, EPI-factor: 122 and FoV: 240 mm. Parallel imaging was performed with a generalised auto-calibrating partially parallel acquisition (GRAPPA), reconstruction algorithm and an acceleration factor of 2. High-resolution 3D T1-weighted MPRAGE images were acquired with full head-coverage with the following parameters: TR=2200 ms, TE=2.28 ms, T1=994 ms, 208 slices, isotropic voxel size=1 mm^3^, flip angle=8° and FoV: 260 mm. Consistent image quality was ensured by a semi-automated quality control procedure.

***MRI quality control assessment***

The quality of diffusion-weighted MRI data was assessed both visually and automatically. A single-subject report was obtained using the Quality Assessment for DMRI (QUAD) method, and a group statistic for motion and distortion corrections was evaluated with the Study-wise Quality Assessment for DMRI (SQUAD) method (<https://git.fmrib.ox.ac.uk/matteob/eddy_qc_release>) (Bastiani et al., 2019)

For all participants, diffusion-weighted MRI presented good quality, and no artifacts compromised data quality. For motion quantification, at a group level, the measured average absolute motion was 0.275±0.27 mm (mean ± standard deviation), and relative motion was 0.0927±0.039 mm; distribution plots are shown in Supplementary Figure 1 (Figure S1).

***Diffusion-weighted MRI preprocessing***

Diffusion-weighted images were skull-stripped using the FSL-bet function. Image denoising was performed with the following MRtrix3 functions: dwidenoising, mrdegibbs and dwibiascorrect. Eddy-current effects, and signal drop-out were corrected using FSL-eddy. Diffusion tensor maps were generated using the FSL-dtifit function, including fractional anisotropy (FA), mean diffusivity (MD), axial diffusivity (AD) and radial diffusivity (RD).

***Reading the Mind in the Eyes (RMET)***

“Reading the Mind in the Eyes” Test (RMET) (Baron-Cohen et al., 2001), a widely used computer-based behavioural task in ToM investigations, was used to evaluate affective ToM ability. The task presents 28 images of multiple expressions of different individuals’ eyes with four adjectives and participants are asked to select the one that best describes what the person is feeling as quickly as possible. The task also includes a control condition, where participants select the age and gender of individuals’ eyes. The two conditions were counterbalanced across participants. Response accuracy and reaction times (RT) were recorded.

**References**

Bastiani M, Cottaar M, Fitzgibbon SP, Suri S, Alfaro-Almagro F, Sotiropoulos SN, Jbabdi S, Andersson JLR (2019). Automated quality control for within and between studies diffusion MRI data using a non-parametric framework for movement and distortion correction. *Neuroimage*, 184, 801-812.

Smith SM, Jenkinson M, Johansen-Berg H, Rueckert D, Nichols TE, Mackay CE, Watkins KE, Ciccarelli O, Cader ZM, Matthews PM, Behrens TE (2006). Tract-based spatial statistics: Voxel-wise analysis of multi-subject diffusion data. *Neuroimage*, 31, 1487-505.

**Table S1** Group differences in tract measurements of the Uncinate Fasciculus (UF), Anterior Thalamic Radiation (ATR), Inferior Longitudinal Fasciculus (ILF) and Inferior Fronto-Occipital Fasciculus (IFOF) tracts

| **Tract**  **Measurement^a^** | **Childhood Maltreatment group (n=34)** | |  | **Peer Victimisation group (n=35)** | |  | **Comparison group**  **(n=38)** | |  | **Group Comparisons^b,c,d^** | | | | | | | | |
| --- | --- | --- | --- | --- | --- | --- | --- | --- | --- | --- | --- | --- | --- | --- | --- | --- | --- | --- |
|  |  |  |  |  |  |  |  |  |  | ***CM vs C*** | | | ***PV vs C*** | | | ***CM vs PV*** | | |
|  | **Mean** | **SD** |  | **Mean** | **SD** |  | **Mean** | **SD** |  | ***F*(1, 67)** | ***p*** |  | ***F*(1, 68)** | ***p*** |  | ***F*(1, 64)** | ***p*** |  |
| **Right UF** | | |  |  |  |  |  |  |  |  |  |  |  |  |  |  |  |  |
| MD  (x 10^-3^ mm^2^/s) | 0.708 | 0.018 |  | 0.710 | 0.019 |  | 0.712 | 0.015 |  | 2.30 | ns | **-** | 1.34 | ns | **-** | 0.29 | ns | **-** |
| RD  (x 10^-3^ mm^2^/s) | 0.556 | 0.024 |  | 0.560 | 0.027 |  | 0.561 | 0.018 |  | 6.53 | 0.013 | CM < C | 6.14 | 0.016 | PV < C | 2.76 | ns | **-** |
| AD  (x 10^-3^ mm^2^/s) | 0.101 | 0.024 |  | 0.101 | 0.019 |  | 0.102 | 0.022 |  | 0.06 | ns | **-** | 1.34 | ns | **-** | 3.85 | (0.05) | (CM > PV) |
| **Left UF** | | |  |  |  |  |  |  |  |  |  |  |  |  |  |  |  |  |
| MD  (x 10^-3^ mm^2^/s) | 0.694 | 0.020 |  | 0.699 | 0.021 |  | 0.699 | 0.018 |  | 1.45 | ns | **-** | 0.41 | ns | **-** | 1.34 | ns | **-** |
| RD  (x 10^-3^ mm^2^/s) | 0.545 | 0.027 |  | 0.550 | 0.031 |  | 0.548 | 0.023 |  | 1.38 | ns | **-** | 1.52 | ns | **-** | 2.01 | ns | **-** |
| AD  (x 10^-3^ mm^2^/s) | 0.100 | 0.026 |  | 0.100 | 0.021 |  | 0.101 | 0.021 |  | 0.001 | ns | **-** | 0.34 | ns | **-** | 0.11 | ns | **-** |
| **Right ATR** | | |  |  |  |  |  |  |  |  |  |  |  |  |  |  |  |  |
| MD  (x 10^-3^ mm^2^/s) | 0.604 | 0.011 |  | 0.605 | 0.015 |  | 0.608 | 0.012 |  | 0.001 | ns | **-** | 0.68 | ns | **-** | 0.55 | ns | **-** |
| RD  (x 10^-3^ mm^2^/s) | 0.475 | 0.014 |  | 0.480 | 0.018 |  | 0.481 | 0.015 |  | 0.49 | ns | **-** | 0.81 | ns | **-** | 0.001 | ns | **-** |
| AD  (x 10^-3^ mm^2^/s) | 0.871 | 0.021 |  | 0.864 | 0.020 |  | 0.871 | 0.017 |  | 0.09 | ns | **-** | 1.01 | ns | **-** | 2.27 | ns | **-** |
| **Left ATR** | | |  |  |  |  |  |  |  |  |  |  |  |  |  |  |  |  |
| MD  (x 10^-3^ mm^2^/s) | 0.588 | 0.012 |  | 0.588 | 0.012 |  | 0.591 | 0.013 |  | 1.49 | ns | **-** | 0.11 | ns | **-** | 0.29 | ns | **-** |
| RD  (x 10^-3^ mm^2^/s) | 0.459 | 0.016 |  | 0.463 | 0.016 |  | 0.464 | 0.017 |  | 0.97 | ns | **-** | 0.001 | ns | **-** | 0.54 | ns | **-** |
| AD  (x 10^-3^ mm^2^/s) | 0.852 | 0.018 |  | 0.844 | 0.017 |  | 0.852 | 0.018 |  | 0.66 | ns | **-** | 0.34 | ns | **-** | 4.70 | 0.034 | CM > PV |
| **Right ILF** | | | | | |  |  |  |  |  |  |  |  |  |  |  |  |  |
| MD  (x 10^-3^ mm^2^/s) | 0.663 | 0.017 |  | 0.669 | 0.019 |  | 0.666 | 0.018 |  | 1.30 | ns | **-** | 3.82 | (0.06) | (PV > C) | 1.16 | ns | **-** |
| RD  (x 10^-3^ mm^2^/s) | 0.483 | 0.021 |  | 0.490 | 0.027 |  | 0.489 | 0.021 |  | 0.61 | ns | **-** | 4.60 | 0.036 | PV > C | 1.38 | ns | **-** |
| AD  (x 10^-3^ mm^2^/s) | 0.102 | 0.024 |  | 0.102 | 0.025 |  | 0.102 | 0.026 |  | 1.37 | ns | **-** | 0.001 | ns | **-** | 0.15 | ns | **-** |
| **Left ILF** | | | | |  |  |  |  |  |  |  |  |  |  |  |  |  |  |
| MD  (x 10^-3^ mm^2^/s) | 0.667 | 0.021 |  | 0.677 | 0.023 |  | 0.675 | 0.019 |  | 0.67 | ns | **-** | 2.74 | ns | **-** | 2.07 | ns | **-** |
| RD  (x 10^-3^ mm^2^/s) | 0.478 | 0.025 |  | 0.489 | 0.031 |  | 0.488 | 0.021 |  | 0.40 | ns | **-** | 4.93 | 0.030 | PV > C | 1.88 | ns | **-** |
| AD  (x 10^-3^ mm^2^/s) | 0.104 | 0.025 |  | 0.105 | 0.024 |  | 0.104 | 0.029 |  | 0.18 | ns | **-** | 0.04 | ns | **-** | 0.55 | ns | **-** |
| **Right IFOF** | | | | | |  |  |  |  |  |  |  |  |  |  |  |  |  |
| MD  (x 10^-3^ mm^2^/s) | 0.654 | 0.015 |  | 0.659 | 0.017 |  | 0.658 | 0.016 |  | 1.21 | ns | **-** | 1.30 | ns | **-** | 0.41 | ns | **-** |
| RD  (x 10^-3^ mm^2^/s) | 0.456 | 0.017 |  | 0.463 | 0.023 |  | 0.462 | 0.018 |  | 0.80 | ns | **-** | 5.12 | 0.027 | PV > C | 1.25 | ns | **-** |
| AD  (x 10^-3^ mm^2^/s) | 0.105 | 0.025 |  | 0.105 | 0.023 |  | 0.105 | 0.029 |  | 0.13 | ns | **-** | 1.35 | ns | **-** | 0.11 | ns | **-** |
| **Left IFOF** | | | | | |  |  |  |  |  |  |  |  |  |  |  |  |  |
| MD  (x 10^-3^ mm^2^/s) | 0.651 | 0.017 |  | 0.659 | 0.017 |  | 0.658 | 0.018 |  | 0.18 | ns | **-** | 1.73 | ns | **-** | 1.63 | ns | **-** |
| RD  (x 10^-3^ mm^2^/s) | 0.444 | 0.021 |  | 0.456 | 0.026 |  | 0.453 | 0.018 |  | 0.97 | ns | **-** | 5.50 | 0.022 | PV > C | 5.15 | 0.027 | CM < PV |
| AD  (x 10^-3^ mm^2^/s) | 0.106 | 0.027 |  | 0.106 | 0.024 |  | 0.106 | 0.028 |  | 0.07 | ns | **-** | 0.64 | ns | **-** | 1.65 | ns | **-** |

^a^ MD=mean diffusivity; RD=radial diffusivity; AD=axial diffusivity.

^b^ CM=childhood maltreatment group; PV=peer victimisation group; C=comparison group.

^c^ Group differences in MD, RD, AD values were conducted with number of recent stressful life events, age onset and duration of early-life stress exposure score as covariates.

^d^ The values in parentheses are marginally statistically significant.

**Fig. S1** Study-wise quality assessment for DMRI (SQUAD) statistic output


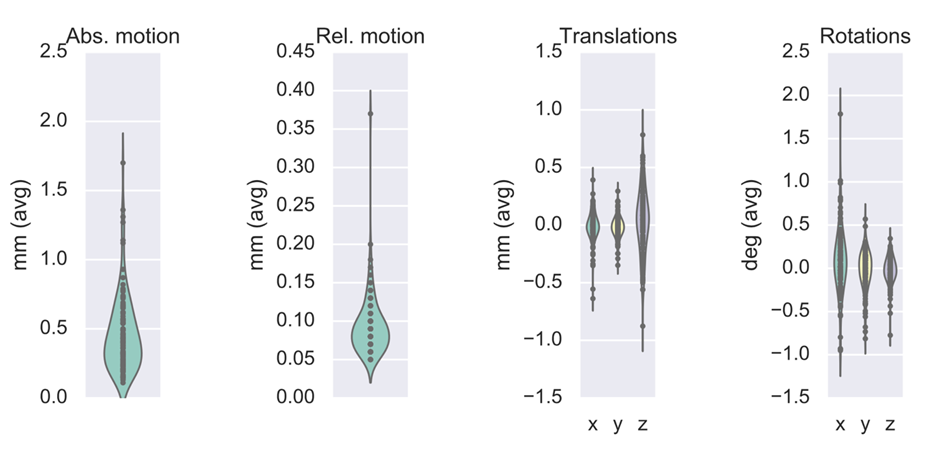


Note: Abs. motion: absolute motion. Rel. motion: relative motion.

**Fig. S2** Tractographic ROIs used for the Uncinate Fasciculus (UF), Anterior Thalamic Radiation (ATR), Inferior Longitudinal Fasciculus (ILF) and Inferior Fronto-Occipital Fasciculus (IFOF) tracts


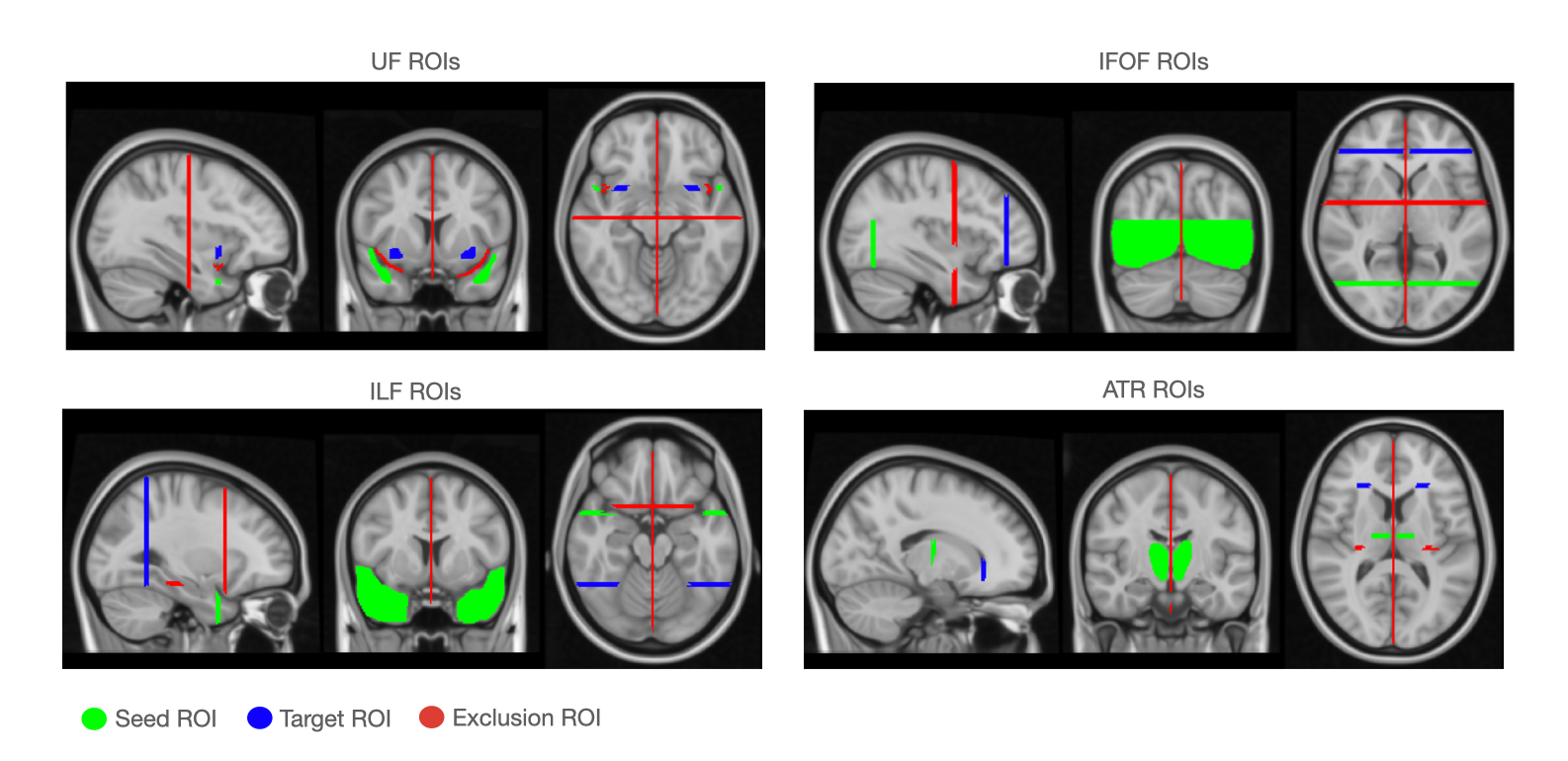


**Fig. S3** Along-tract segment contours are displayed onto the FSL-HCP1065 map in the MNI-152 space. This illustration aims to generalise the along-tract procedure evaluated independently for each subject using the Laplacian operator. The along-tract segment order increases from the anteriorly located segments to the most posteriors. For each tract, a 3D scatter plot shows the voxel localisation coloured according to the respective along-tract segment number. Coordinates (x, y, and z) are reported according to voxel localisation in the MNI-152 space (2 mm resolution)

UF: Uncinate Fasciculus; ATR: Anterior Thalamic Radiation; ILF: Inferior Longitudinal Fasciculus; and IFOF: Inferior Fronto-Occipital Fasciculus.


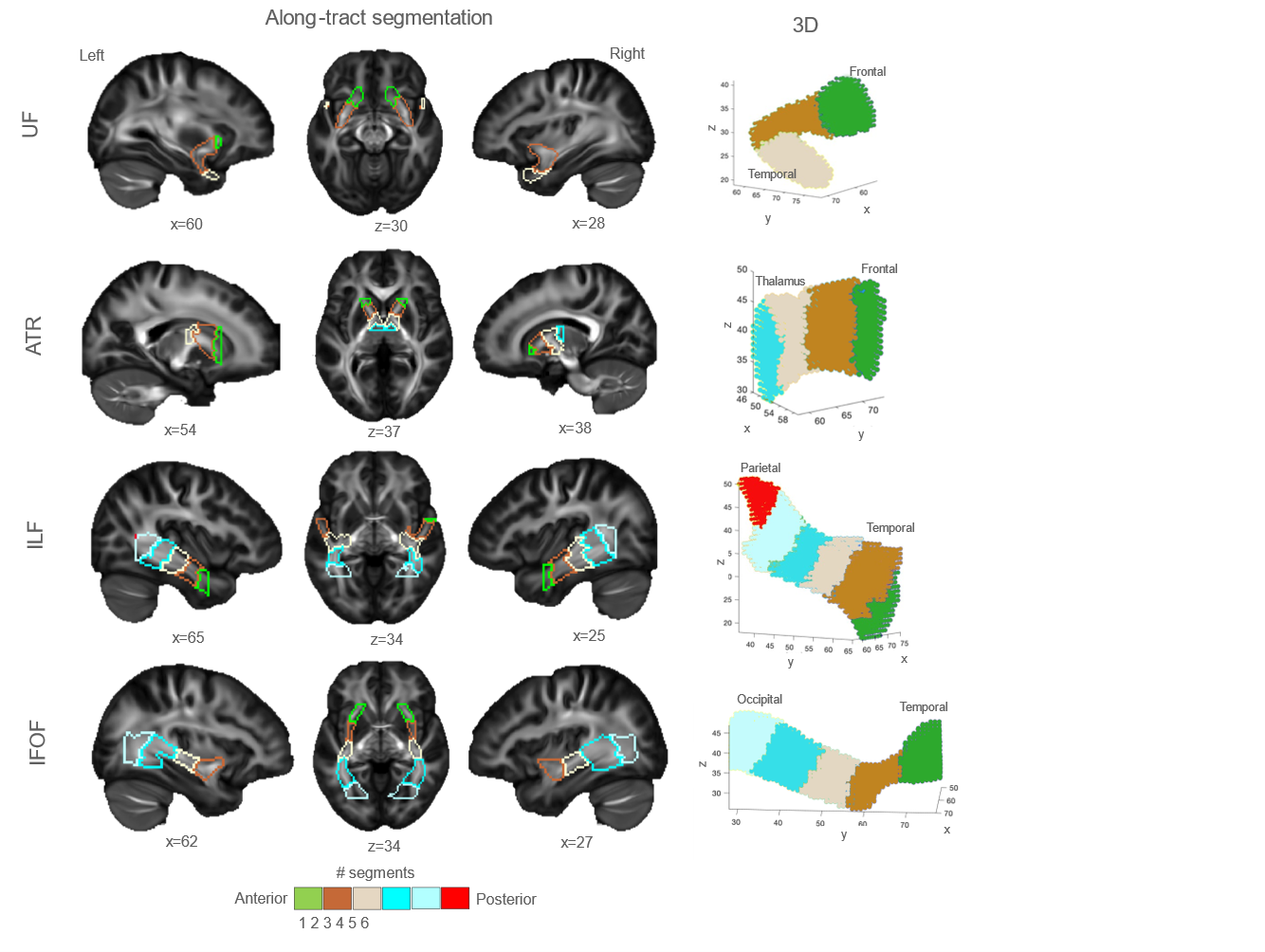


**Fig. S4** Scatterplots of negative correlations between right anterior thalamic radiation (ATR) tract volume and **(a)** CTQ emotional abuse within the childhood maltreatment group, **(b)** CTQ total score within the childhood maltreatment group and **(c)** BAI within the comparison group

**(a) (b) (c)**


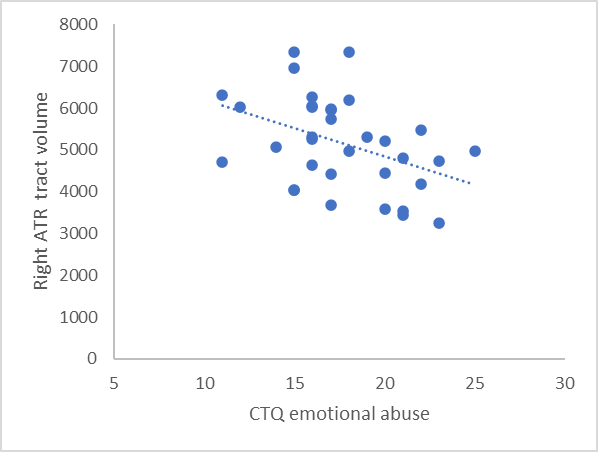

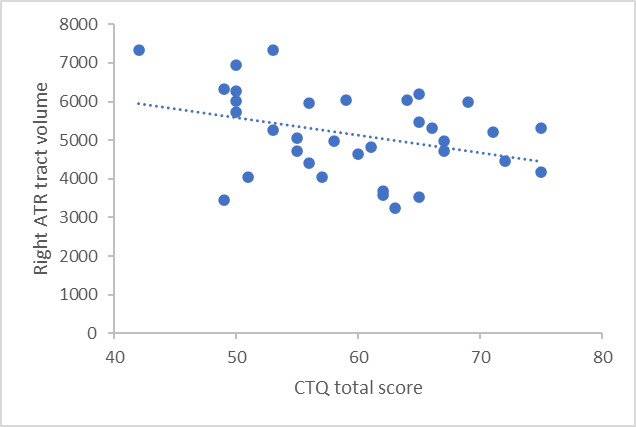

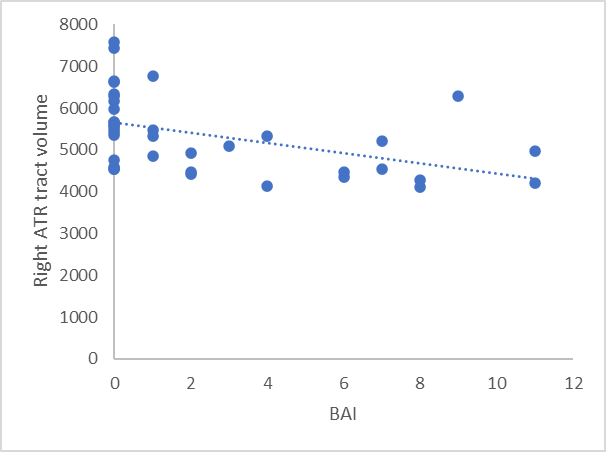


**Fig. S5** Scatterplots of positive correlations between percentage of correct responses in RMET emotional-state condition and **(a)** left anterior thalamic radiation (ATR) FA within the childhood maltreatment group, **(b)** left inferior fronto-occipital fasciculus (IFOF) FA within the peer victimisation group and **(c)** right uncinate fasciculus (UF) FA within the peer victimisation group

**(a) (b) (c)**


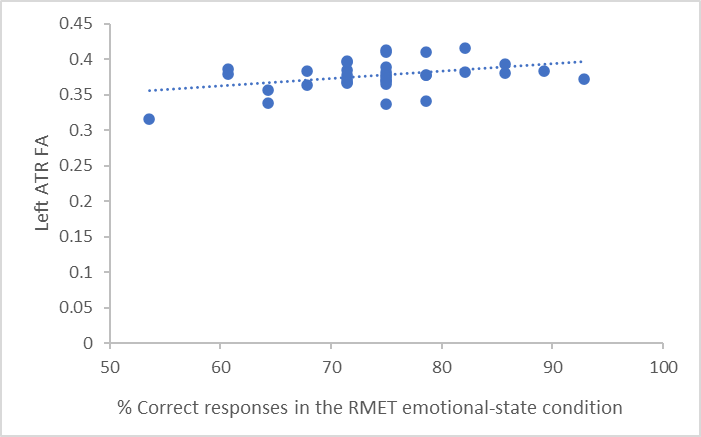

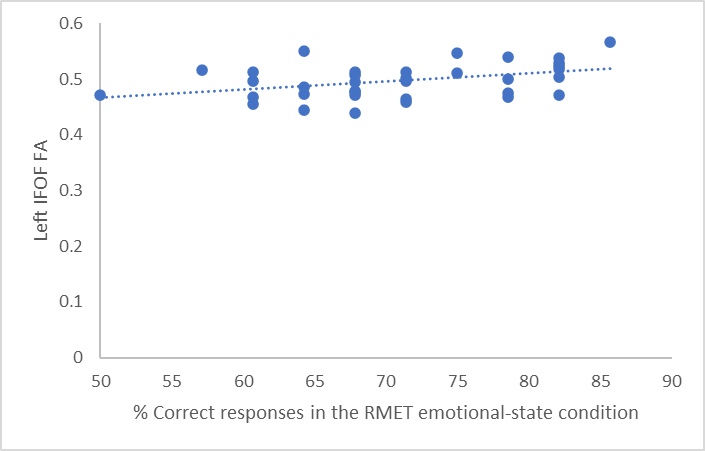

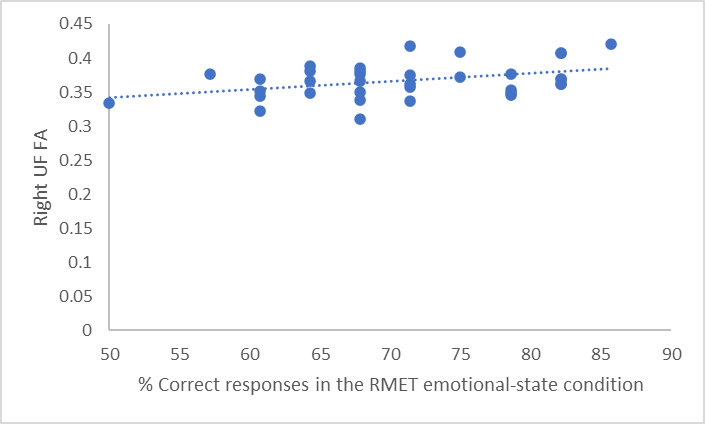


**Fig. S6** Scatterplots of **(a)** negative correlation between left inferior fronto-occipital fasciculus (IFOF) FA and SDQ emotional problems within the comparison group and **(b)** negative correlation between right anterior IFOF FA and SDQ peer problems within the peer victimisation group

**(a) (b)**


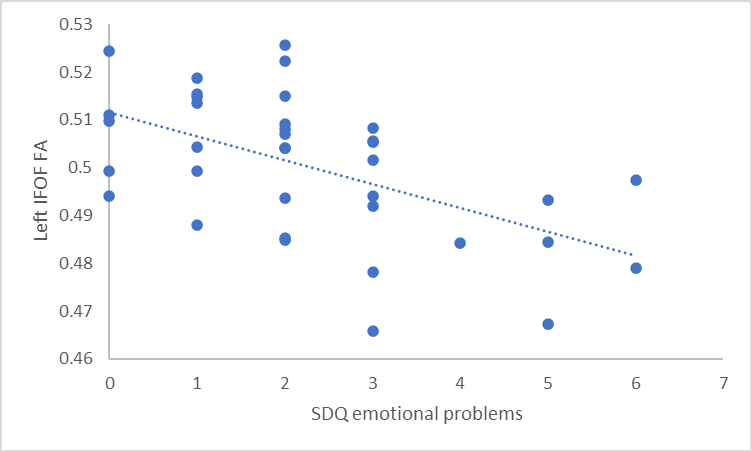

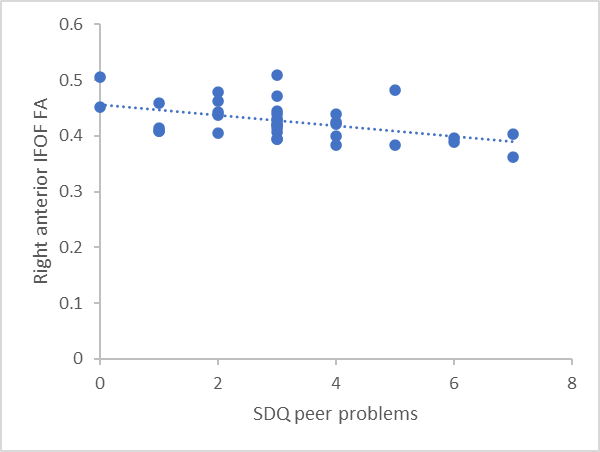

Supplement: Supplementary file 1 — Supplementary Material 1. [file 12888_2024_5759_MOESM1_ESM.docx]
